# Physiological Stress Response and Oxidative Status in Tambaqui (*Colossoma macropomum*) Fed Diets Supplemented with Selenium

**DOI:** 10.3390/biology13120959

**Published:** 2024-11-22

**Authors:** Celma Maria Ferreira, Valéria Dornelles Gindri Sinhorin, Márcio Aquio Hoshiba, Janessa Sampaio de Abreu

**Affiliations:** 1 Animal Science Graduate Program, Federal University of Mato Grosso (UFMT), Av. Fernando Corrêa da Costa, 2367, Boa Esperança, Cuiabá 78060-900, MT, Brazil; celma.ferreira@unesp.br; 2 Department of Chemistry, Institute of Chemistry, Federal University of Mato Grosso (UFMT), Av. Fernando Corrêa da Costa, 2367, Boa Esperança, Cuiabá 78060-900, MT, Brazil; valeria.sinhorin@ufmt.br; 3 Faculty of Agronomy and Animal Science, Federal University of Mato Grosso (UFMT), Av. Fernando Corrêa da Costa, 2367, Boa Esperança, Cuiabá 78060-900, MT, Brazil; tokudazoo@gmail.com

**Keywords:** cortisol, fish physiology, hydroxy-selenomethionine, oxidative stress

## Abstract

The present study aimed to evaluate the physiological responses to transport stress in juvenile tambaqui (*Colossoma macropomum*) fed a diet supplemented with hydroxy-selenomethionine (OH-SeMet; Selisseo^®^, Adisseo) and determine through stress biomarkers whether selenium supplementation could reduce the impact of transport stress on tambaqui resilience. Juvenile fish (15.71 ± 1.90 g) were randomly distributed into five experimental groups; one fed a basal diet without inclusion of OH-SeMet (0.0 mg Se/kg) and four other groups fed supplemented diets with selenium in the form of OH-SeMet at concentrations of 0.3, 0.6, 0.9, and 1.2 mg Se/kg over 75 days. After this period, the fish were transported for 4 h in plastic bags (60 L), and sampling took place before transport (baseline), immediately after transport, and 24 h later. The effects of dietary OH-SeMet in tambaqui challenged by transport were studied in terms of antioxidative status (superoxide dismutase, catalase, glutathione peroxidase, glutathione s-transferase, and reduced glutathione); stress markers (serum cortisol, blood glucose, ionic and hematological parameters), and immunological status (respiratory burst activity and total protein, A/G ratio). Following transport, we observed an elevation in cortisol and blood glucose concentrations, accompanied by hemoconcentration, as evidenced by elevated hematocrit, erythrocyte count, hemoglobin concentration, and mean corpuscular hemoglobin concentration (MCHC). Additionally, there was a decrease in immune response, indicated by reduced respiratory activity of leukocytes and an osmotic imbalance, which culminated in hypochloremia, increased plasma protein concentration, and a rise in red blood cell volume, all of which were measured 24 h after the stressor was applied. Twenty-four hours after transport, the antioxidant defense system was activated, and no recovery of homeostasis was observed for most of the parameters analyzed. The dietary inclusion of selenium did not alleviate the hormonal, metabolic, hematological, and ionic changes caused by transport, but at a concentration of 0.9 mg Se/kg, it was able to increase the activity of enzymes related to oxidative stress (catalase and superoxide dismutase, mainly) in tambaqui juveniles submitted to this challenge.

## Simple Summary

In this study, tambaqui (*Colossoma macropomum*) juveniles were treated with diets containing selenium for 75 days and then were transported in plastic bags for 4 h and had their physiological responses evaluated to verify whether selenium could mitigate the effects of stress caused by transport. The results showed the dietary inclusion of selenium did not alleviate the hormonal, metabolic, hematological, and ionic changes caused by transport, but at a concentration of 0.9 mg Se/kg, it was able to increase the activity of enzymes related to oxidation in tambaqui juveniles submitted to this challenge. In summary, these results demonstrate the beneficial effects of selenium supplementation on the antioxidant status of tambaqui juveniles, especially in the presence of stressors.

## 1. Introduction

In fish farming, fish are continually exposed to routine handling, such as capture and transportation. Although necessary, these procedures cause stress in animals, and over the years, several studies have described the physiological responses of fish to stressors typical of aquacultural practices [1,2,3,4]. Among the changes caused by stress is the increased mobilization and excretion of minerals and vitamins from tissues, which can result in an increased insertion of minerals. Moreover, stressors such as transport generate oxidative stress and reduce the antioxidant defense of animals in vivo, causing oxidative damage and interfering with plasma concentrations of antioxidant minerals [5].

A nutritionally balanced aquatic diet can help neutralize the deleterious effects of stressors and improve productivity, and the addition of microelements is important to ensure the nutritional value of aquatic foods [6]. In this sense, selenium has been considered an excellent nutrient essential for the improvement of aquaculture products precisely because of its crucial role in the biological functions of the fish body, encompassing roles as an immunostimulant, antioxidant, and metabolic stimulator [7].

It is documented that dietary selenium administration increased growth performance and immunological function in various fish species, like *Cyprinus carpio* [8], *Oncorhynchus mykiss* [9], *Oreochromis niloticus* [10], and Brazilian freshwater fish species, such as *Piaractus mesopotamicus* [11,12] and *Colossoma macropomum* [13].

Selenium acts like a cofactor of the glutathione peroxidase (GPx; antioxidant enzyme), playing a relevant role in oxidative stress, which makes this mineral one of the most tested in reducing this stress in fish due to its antioxidant action [14]. However, most investigations have invested in the response to oxidative stress, relating the beneficial effects of Se supplementation, while few evaluate this effect on physiological changes resulting from the activation of the Hypothalan–Pituitary–Interrenal axis when fish are exposed to stressors. Effects of selenium on inhibiting cortisol inhibition were observed in *Megalobrama amblycephala* following exposure to nitrite [15]. In *Sparus aurata* fed 0.2 mg of hydroxy-selenomethionine/kg of feed, there was a significant reduction in plasma cortisol after 2 h of acute stress [16]. Rider et al. [17] found that dietary selenium supplementation was necessary to meet the requirements of *Oncorhynchus mykiss* during physical stress.

Tambaqui (*Colossoma macropomum*) is a relevant species for fish farming in South America, standing out among native fish as the most produced in Brazil [18]. For this species, responses to transport stress are well documented [19,20,21,22,23], but none of the studies investigated the effect of selenium on minimizing stress during transport handling and modulating the innate immune and antioxidant systems.

Understanding stress responses during transport is crucial for developing effective management practices. Considering that immunonutrients could mitigate the physiological stress response, this study aimed to evaluate the physiological responses to transport stress in juvenile tambaqui (*Colossoma macropomum*) fed a diet supplemented with hydroxyselenomethionine and determine, through hormonal, metabolic, ionic, and hematological indicators, whether selenium administration in the diet could minimize the effects of transport stress on tambaqui resilience.

## 2. Material and Methods

### 2.1. Experimental Design

Juvenile tambaqui (*Colossoma macropomum*) (15.71 ± 1.90 g) were distributed in 15 experimental 150 L polyethylene tanks (13 fish/tank) in a recirculation system supplied with a continuous flow of water and constant aeration. Before starting the experiment, the fish were acclimatized to laboratory conditions and fed the basal diet (38% crude protein) formulated considering the nutritional requirements described for the species [24], using raw materials frequently used in the animal feed industry and a vitamin and mineral complex prepared without a selenium source (Premix Nutrepharm, Cuiabá, MT, Brazil).

The ingredients were mechanically ground and mixed manually, with the basal diet supplemented with selenium in the form of hydroxy-selenomethionine (OH-SeMet) (OH-SeMet) (Selisseo^®^, Adisseo France S.A.S., Antony, France) at concentrations of 0, 0.3, 0.6, 0.9, and 1.2 mg Se/kg, determined based on prior research conducted with native fish species [11,12]. The mixture was moistened with distilled water and then pelletized using a meat grinder (CAF, Rio Claro, São Paulo, Brazil). After, the diets were dried in a forced circulation oven (SOLAB-102) at 55 °C for ten hours and then allowed to cool to room temperature. The experimental diets were stored in a −20 °C freezer until feeding. The final selenium concentrations in the experimental diets were 0.10, 0.34, 0.65, 0.91, and 1.26 mg Se/kg, respectively, as determined by graphite furnace-coupled atomic absorption spectrometry (HG-AAS).

The experiment was conducted using a completely randomized design with five treatments (0.0, 0.3, 0.6, 0.9, and 1.2 mg Se/kg of feed) with three replicates for each treatment. The experimental diets were provided to the fish twice daily (09:00 a.m. and 15:00 p.m.) until apparent satiety for 75 days. At the end of this period, the fish in the experimental tanks were fasted for 24 h to empty the gastrointestinal tract. They were then randomly removed from each tank, distributed in plastic bags (60 L, 12 fish per bag) containing water and oxygen, and transported for 4 h in a utility vehicle. After transport, fish were returned to the tanks from which they were taken for recovery.

Sampling occurred before transport (BT, *n* = 15), immediately after transport (AT, *n* = 45), and 24 h after transport (24 h AT, *n* = 45). At each time, the animals were fasted for 12 h before being captured (*n* = 9 per treatment), anesthetized with 30 mg of eugenol per liter of water, and blood was collected by puncturing the tail vein to assess hematological and immunological parameters. Subsequently, they were sacrificed by sectioning the spinal cord to remove the liver. Liver tissue samples were stored at −80 °C for the analysis of enzymatic and non-enzymatic antioxidants.

At each sampling time, limnological parameters were monitored, such as water temperature and dissolved oxygen (YSI Pro 20 digital oximeter, Yellow Springs, OH, USA), pH (Quimis^®^ digital bench pH meter—Q400BD, Diadema, São Paulo, Brazil), alkalinity (titration from the methyl orange indicator solution), and non-ionized ammonia, calculated according to Emerson et al. [25].

### 2.2. Hematological, Biochemical, Immunological, and Antioxidant System Analyses

Blood aliquots were collected with and without anticoagulants (heparin and EDTA). In the heparinized blood, leukocyte respiratory burst activity was assessed according to the protocol outlined by Sahoo et al. [26]. The following variables were measured in the blood collected with EDTA: hemoglobin concentration (Labtest^®^ commercial kit, Lagoa Santa, Minas Gerais, Brazil), hematocrit (microcentrifuge with glass capillary tubes), and erythrocyte number (Neubauer chamber), used to determine mean corpuscular volume (MCV = Hematocrit/Erythrocyte number × 10) and mean corpuscular hemoglobin concentration (MCHC = Hemoglobin rate × 100/Hematocrit). The differential leukocyte count was performed on blood smears stained with May Grünwald–Giemsa Wright (MGGW), according to Tavares-Dias and Moraes [27].

The blood without anticoagulant was centrifuged (clinical centrifuge 80-2B) for 10 min at 3000 rpm to separate the serum and plasma. Blood glucose levels in the plasma were measured using a commercial kit (Labtest^®^). Cortisol levels in the serum were assessed using an enzyme immunoassay based on ELISA (DRG^®^ kit) and the concentration of chloride, total proteins, and albumin using a commercial kit (Labtest^®^). The globulin concentration was estimated through the difference between the total protein concentration and albumin concentration, and the albumin–globulin ratio (A:G) was obtained by dividing the albumin value by the globulin value.

Biochemical parameters were evaluated in liver tissue, including the activity of superoxide dismutase (SOD) enzymes according to Misra and Fridovich [28]; catalase (CAT) and glutathione peroxidase (GPX) according to Nelson and Kiesow [29] and Paglia and Valentine [30], respectively; glutathione-s-transferase (GST) according to Habig et al. [31], and the concentration of reduced glutathione (GSH) according to the methodology proposed by Sedlack and Lindsay [32].

### 2.3. Statistical Analysis

The data were subjected to the normality and homogeneity of variances test. When they presented normal distribution, they were analyzed using parametric statistics using the SAS version 9.0 (Statistical Analysis System) program. When the data did not present a normal distribution, they were subjected to logarithmic transformation (log x) and submitted to analysis of variance (ANOVA) using parametric statistics.

A completely randomized design (CRD) with a factorial design of 5 × 3 (treatments × sampling times) was employed. The data were analyzed by a two-way analysis of variance (ANOVA), with treatments (0, 0.3, 0.6, 0.9, and 1.2 mg Se/kg) and sampling times (before transport, immediately after transport, and 24 h after transport) as the factors. For the parameters in which the interaction between the factors was not significant (*p* ≥ 0.05), such as cortisol, blood glucose, chloride, hematocrit, erythrocyte number, hemoglobin concentration, MCHC, MCH, leukocyte respiratory burst activity, total proteins, globulin, and A:G ratio, the effects of treatments and sampling times were compared separately (one-way analysis). For those in which there was a significant interaction (*p* < 0.05) between the factors (MCV, percentage of leukocytes, albumin, and antioxidant enzymes), the degrees of freedom of the treatments were broken down within each sampling time.

When F values indicated significance (*p* < 0.05), means were compared using the Tukey test. When the ANOVA assumptions were not met after transformation, the Kruskal–Wallis’s non-parametric test was used, followed by the Dunn’s test.

All statistical tests were applied at a 95% confidence level (*p* < 0.05) and performed using R software programs, version 4.4.2; SAS version 9.0 (Statistical Analysis System) and Graph Pad Prism 5 (Graph Pad Software Inc., San Diego, CA, USA).

## 3. Results

### 3.1. Water Analysis

Regardless of the treatments, there was a significant decrease in oxygen, temperature, pH, and alkalinity levels immediately after transport. The levels of toxic ammonia (NH_3_) were not affected by this procedure (Table 1).

### 3.2. Hematological, Biochemical, Immunological, and Antioxidant System Analyses

There was no significant interaction between selenium concentrations and sampling time for blood glucose, serum cortisol, and chloride levels, hematological parameters (except MCV), oxidative burst, and profile protein (except albumin). Thus, for these indicators, the results are presented, showing the effects of different treatments (regardless of sampling time) and different sampling times (regardless of different treatments).

The serum cortisol level was lower (*p* = 0.0001) in tambaqui fed with 1.2 mg Se/kg when compared to fish fed with 0.3 and 0.9 mg Se/kg. Regardless of selenium supplementation in the diet, there was an increase in serum cortisol upon arrival of transport (*p* < 0.0001) without recovery of initial values up to 24 h after carrying out this management. Likewise, the blood glucose of the fish increased upon arrival (*p* < 0.0001), without recovery of baseline values 24 h after the procedure (Table 2).

**Figure 1:**
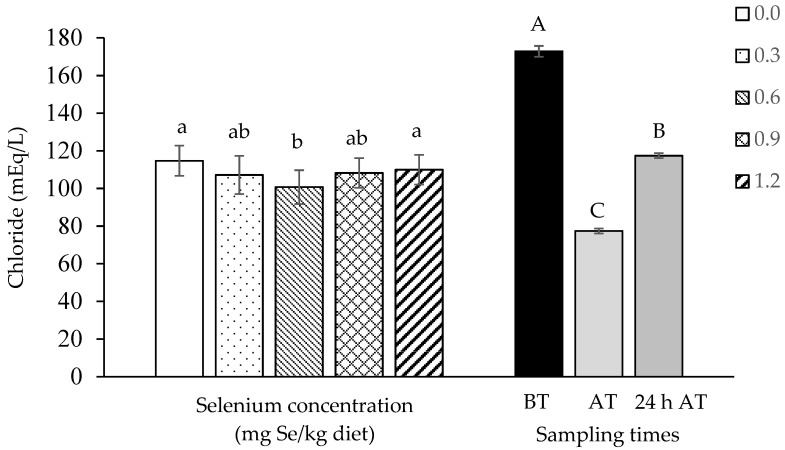
Serum chloride from tambaqui (*Colossoma macropomum*) supplemented with selenium in the diet (hydroxy-selenomethionine; 0.0, 0.3, 0.6, 0.9, and 1.2 mg Se/kg) and submitted to transport stress. Different lowercase letters show significantly different data between treatments and capital letters between times (ANOVA; Tukey test; *p* < 0.05). BT: before transport; AT: after transport; 24 h AT: 24 h after transport.

Serum chloride levels were lower (*p* = 0.0278) in tambaqui supplemented with 0.6 mg Se/kg in relation to fish fed diets not containing selenium (0.0 mg/kg) and containing the highest inclusion (1.2 mg/kg). In all treatments, there was a significant drop in chloride immediately after transport (*p* < 0.0001), with no recovery within 24 h (Figure 1).

Hematocrit, mean corpuscular hemoglobin concentration (MCHC), and hemoglobin increased (*p* < 0.0001) upon arrival of transport, regardless of the concentration of selenium in the diet (Table 3), with recovery to the initial condition within 24 h. There was a decrease in the total number of erythrocytes and an increase in mean corpuscular hemoglobin (MCH) (*p* < 0.0001) 24 h after transport (Table 3).

**Figure 2:**
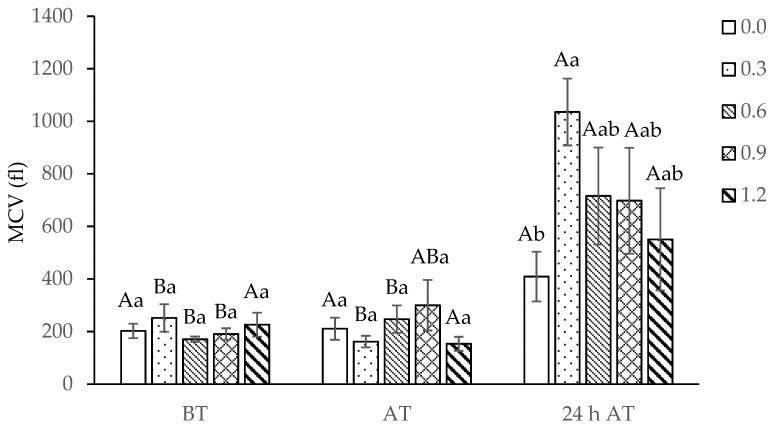
Mean corpuscular volume (MCV) of tambaqui (*Colossoma macropomum*) supplemented with selenium in the diet (hydroxy-selenomethionine; 0.0, 0.3, 0.6, 0.9, and 1.2 mg Se/kg) and submitted to transport stress. Different capital letters show significantly different data between each treatment at different times and lower-case letters between treatments within each time (ANOVA; Tukey test; *p* < 0.05). BT: before transport; AT: after transport; 24 h AT: 24 h after transport.

There was a significant interaction between treatments and sampling times for MCV (*p* = 0.0229), and the treatment results were broken down within each time. The MCV increased in tambaqui fed with up to 0.9 mg of Se/kg within 24 h after transport, and this increase was significant in fish supplemented with 0.3 mg Se/kg in relation to 0.0 mg of Se/kg selenium (Figure 2).

**Figure 3:**
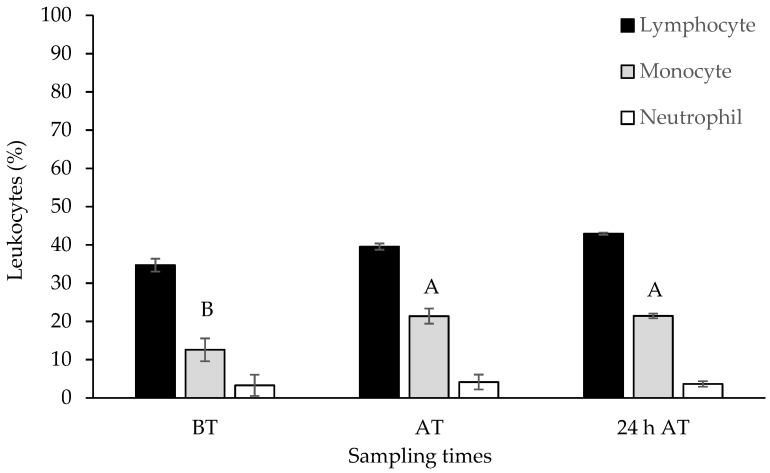
Results obtained from lymphocytes, monocytes, and neutrophils of tambaqui (*Colossoma macropomum*) supplemented with selenium in the diet (hydroxy-selenomethionine; 0.0, 0.3, 0.6, 0.9, and 1.2 mg Se/kg) and submitted to transport stress. Each bar represents the mean ± standard error of all treatments. Different capital letters show significantly different data between sampling times (ANOVA; Tukey test; *p* < 0.05). For statistical analysis, the data were log (x) transformed. BT: before transport; AT: after transport; 24 h AT: 24 h after transport.

Data obtained from leukocyte analysis showed a greater presence of lymphocytes (35 to 43%), followed by monocytes (13 to 21%) and neutrophils (3 to 4%), and the percentage did not change by the inclusion of selenium in the diet. Transport affected the percentage of monocytes, which increased significantly upon arrival, without recovery within 24 h of transport (Figure 3).

**Figure 4:**
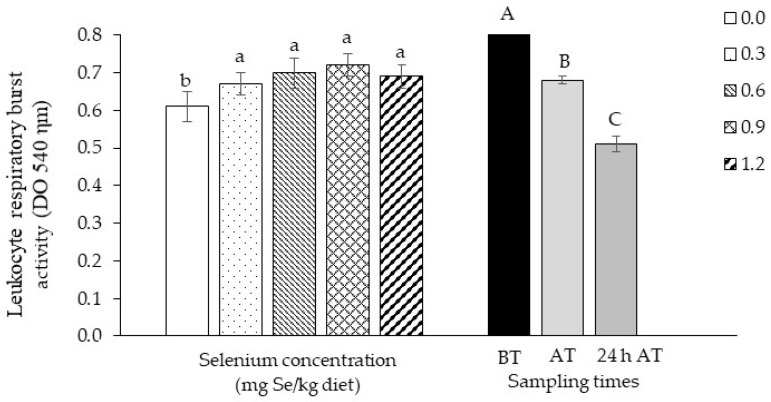
Results obtained from leukocyte respiratory burst activity of tambaqui (*Colossoma macropomum*) supplemented with selenium in the diet (hydroxy-selenomethionine; 0.0, 0.3, 0.6, 0.9, and 1.2 mg Se/kg) and submitted to transport stress. Different lowercase letters show significantly different data between treatments and capital letters between times (ANOVA; Tukey test; *p* < 0.05). BT: before transport; AT: after transport; 24 h AT: 24 h after transport.

Fish fed with selenium showed greater leukocyte respiratory activity. Transport caused a decrease (*p*< 0.0001) in this activity up to 24 h after its completion (Figure 4).

The different concentrations of selenium in the diet did not affect the levels of total protein and globulin, but transport influenced these parameters, with an increase (*p* < 0.0001) on arrival, without recovery of baseline values within 24 h. A lower A:G ratio was observed in tambaqui fed with 0.6 mg Se/kg in relation to other groups (0.3 and 0.9 mg Se/kg) (Table 4).

**Figure 5:**
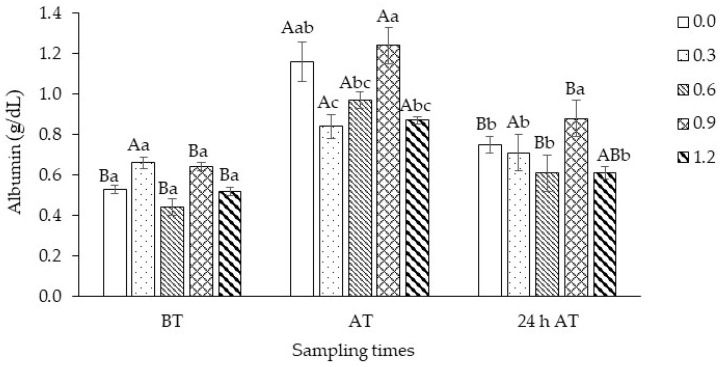
Albumin from tambaqui (*Colossoma macropomum*) supplemented with selenium in the diet (hydroxy-selenomethionine; 0.0, 0.3, 0.6, 0.9, and 1.2 mg Se/kg) and submitted to transport stress. Different capital letters show significantly different data between each treatment at different times and lower-case letters between treatments within each time. (ANOVA; Tukey test; *p* < 0.05). BT: before transport; AT: after transport; 24 h AT: 24 h after transport.

There was a significant interaction (*p* = 0.0010) between treatments and sampling times for albumin, and the treatment results were broken down within each time. Except for fish supplemented with 0.3 mg Se/kg, there was an elevation in albumin concentration upon arrival, with recovery within 24 h after transport. Among the selenium supplementation levels, albumin was significantly higher in fish fed 0.9 mg Se/kg (Figure 5).

**Figure 6:**
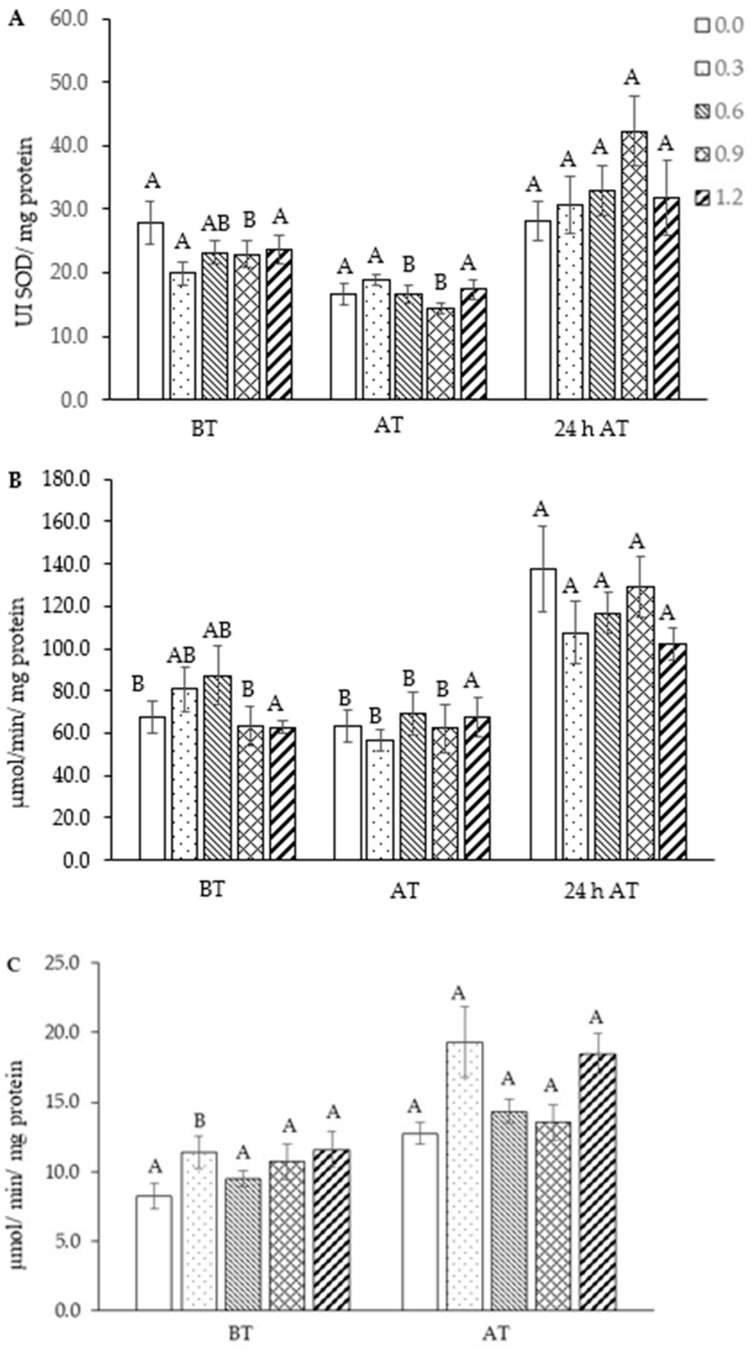
Analysis of enzymatic activities in the liver of tambaqui (*Colossoma macropomum*) supplemented with selenium in the diet (hydroxy-selenomethionine; 0.0, 0.3, 0.6, 0.9, and 1.2 mg Se/kg) and submitted to transport stress. Superoxide dismutase—SOD (**A**), catalase—CAT (**B**), and glutathione peroxidase—GPx (**C**). Different capital letters show significantly different data between treatments at different times (ANOVA; Tukey test; *p* < 0.05). For statistical analysis, data were transformed into log x. BT: before transport; AT: after transport; 24 h AT: 24 h after transport.

**Figure 7:**
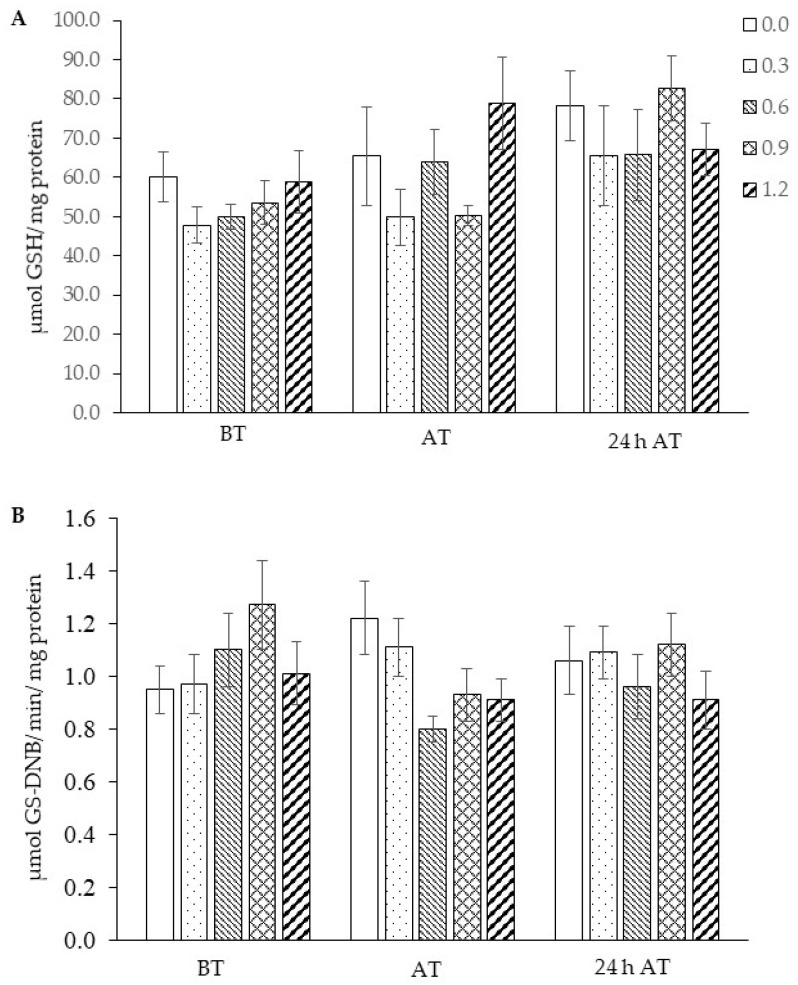
Concentration of reduced glutathione—GSH (**A**) and activity of glutathione s-transferase—GST (**B**) in the liver of tambaqui (*Colossoma macropomum*) supplemented with selenium in the diet (hydroxy-selenomethionine; 0.0, 0.3, 0.6, 0.9, and 1.2 mg Se/kg) and submitted to transport stress. For GSH concentration (ANOVA; Tukey test; *p* < 0.05) and GST activity (Kruskal–Wallis’s Test; Dunn’s test; *p* < 0.05). For statistical analysis, GSH data were transformed into log x.

There was a significant interaction between selenium concentrations and sampling times for SOD (*p* = 0.02936), CAT (*p* = 0.01653), and GPx (*p* = 0.02053) activities; GSH concentration (*p* = 0.02463) and GST activity (*p* = 0.04360) and the treatment results are presented within each time. In tambaqui supplemented with 0.9 mg Se/kg of feed, a significant increase in SOD activity at 24 h after transport was observed (Figure 6A). Likewise, CAT activity was activated within 24 h (0.9 mg Se/kg and without selenium inclusion) (Figure 6B). Although GPx activity was not evaluated 24 h after transport, the results showed greater activity of this enzyme upon arrival of transport only in fish supplemented with 0.3 mg Se/kg (Figure 6C). GSH concentration and GST activity were not changed after treatments and by transport stress (Figure 7A,B).

## 4. Discussion

Transport for 4 h changed the water quality settings, but even with the changes, the values remained within those recommended to produce tropical fish [33], except for dissolved oxygen values, which concentrated around 2.0 mg/L, but without affecting tambaqui survival, which was 100% for fish in all five treatments during transport. Tambaqui (*C. macropomum*) is native to the Amazon basin and adapted to the oxygen variations that occur at certain periods of the year in its natural habitat [34], which explains the fact that the oxygen concentrations observed at the end of transport were not problematic for this species. Similarly, no mortality was observed by Chagas et al. [20] when they subjected tambaqui to transport for 3 h.

Temperature variation is one of the challenges that greatly influences fish transport [35]. The period in which the transport was carried out was affected by cloudy and rainy days, resulting in a milder ambient temperature, which may explain these significantly lower temperatures in the water upon arrival and 24 h after transport.

The excretion of CO_2_ by the fish led to a significant decrease in the pH of the water. This CO_2_ reacts with water to form carbonic acid, leading to increased acidity and a higher concentration of H^+^ ions in the water. This fact is very common when transport is carried out in a closed system, as is the case in this study, in which the fish were transported in plastic bags. Araujo-Lima and Gomes [36] explain that tambaqui tolerates variations in water quality well and can tolerate low pH values (4.0). In this study, the water used for transport came from an artesian well, and the high levels of carbonate and bicarbonate ions guaranteed a functional buffer system so that the increase in hydrogen ion concentration was compensated by the solubilization of CaCO_3_, verified by the decrease in alkalinity upon arrival.

Despite the transport being in a closed system, no significant differences were verified in the levels of toxic ammonia (NH_3_), which may be attributed to the lower pH and temperature values found in this study after transport, resulting in less appearance of toxic ammonia [33].

Cortisol is the predominant corticosteroid in fish, and its plasma levels rise as an initial response to a stressor [2]. This response was demonstrated in this study with an increase in plasma cortisol immediately after transport, regardless of selenium supplementation levels, and 24 h after application of this stressor, cortisol remained high, not returning to the initial condition observed before transport. This result differs from those reported by Gomes et al. [34] and Chagas et al. [20], who verified a recovery in basal cortisol levels for the species 24 h after transport. The stress response of fish to transport seems to vary based on the technique employed, and this difference in recovery time of cortisol may have been a consequence of experiencing stress during capture in tanks, crowding, and handling during loading and discharging.

According to Küçükbay et al. [5], stress leads to a reduction in the antioxidant status of animals, which can lead to an increased requirement for minerals such as selenium. In a previous study that we carried out with this species involving selenium supplementation, it was found that diets containing higher levels of selenium in the form of OH-SeMet (0.9 and 1.2 mg/kg) resulted in increased selenium deposition in the muscle of the tambaqui [13]. This enhanced deposition of selenium in the tissue aids the animal in boosting its capacity to produce selenoproteins, thereby improving its defense mechanisms during stressful conditions [37]. Long et al. [15] reported lower cortisol values in *Megalobrama amblycephala* fed diets supplemented with selenium and exposed to nitrite. Likewise, in *Sparus aurata* fed 0.2 mg of hydroxy-selenomethionine/kg of feed, a significant reduction in plasma cortisol was observed after 2 h of acute stress [16]. In this research, among treatments involving selenium supplementation (0.3 to 1.2 mg Se/kg), fish fed with the highest inclusion (1.2 mg Se/kg) had the lowest cortisol values (62.30 ng/mL), suggesting that selenium may have been mobilized by the fish during transport, contributing to minimizing this stress response.

Blood glucose and chloride levels are commonly utilized as biomarkers to evaluate the impact of stress in fish [3,38]. Hyperglycemia and reduced serum chloride levels are reported in tilapia and carp subjected to acute stressors such as transport and aerial exposure [39,40,41]. The rise in blood glucose levels and the hypochloremia observed in *Colossoma macropomum* following transport were not influenced by selenium supplementation in the diet. This finding corroborates other studies involving this species, where the use of immunostimulants such as β-glucan and probiotics was also ineffective in alleviating transport stress responses [19,20].

Cortisol released in response to transport stress promotes increased oxygen consumption by tissues, thus requiring rapid differentiation and proliferation of the erythrocyte series, which may explain the increase in hematocrit (38.28%) observed in juveniles tambaqui upon arrival. Likewise, the increase in hemoglobin (12.64 g/dL) and MCHC (32.82 g/dL) suggests a greater capacity to transport oxygen through the blood, to meet the increased energy demand caused by transport. Furthermore, transport altered fish homeostasis and negatively influenced the osmoregulation process, verified by the significant increase in VCM and HCM after 24 h of carrying out this handling.

In the species in this study (*Colossoma macropomum*), increases in hematocrit values [34], hemoglobin concentration [42], and MCHC [20] are described after transport, however, there are also reports of no significant changes in these hematological parameters after carrying out this management [19]. Hematological values in fish can vary not only between species but also between individuals of the same species, according to the physiological conditions of the animals, which may explain the different observations found between studies.

It is documented for several exotic and native fish species that dietary supplementation with selenium altered hematological parameters [8,12,43,44,45]. In this work, the volume of red blood cells of tambaqui juveniles fed with the lowest concentrations of selenium (0.3 and 0.6 mg Se/kg) increased in 24 h after transport because of the influx of water through osmosis due to the electrolyte disturbances caused by this procedure.

Selenium is recognized for its immunostimulating properties, actively contributing to the immune system of fish [11]. However, in this study, selenium supplementation in the diet in the form of OH-SeMet did not influence the percentages of lymphocytes, neutrophils, and monocytes in *C. macropomum*. This finding contrasts with observations in exotic species such as *Oncorhynchus mykiss* [17], *Trachinotus blochii* [46], and *Cyprinus carpio* [8], where selenium supplementation led to changes in the percentages of these cell types.

When fish are exposed to a stressful stimulus, lymphopenia (decrease in the number of lymphocytes.) and neutrophilia (rise in the number of neutrophils) are observed [39], which was not verified in this work. Only the percentage of tambaqui monocytes was affected, increasing upon arrival, probably in response to the acute transport stress that favors the mobilization likely as a response to acute transport stress, which promotes the mobilization of defense cells and the distribution of various cell types, according to need [47].

Dietary inclusion of selenium from 0.3 mg/kg increased the respiratory activity of tambaqui leukocytes. This happened because selenium is used as a cofactor in enzymes involved in the metabolization of reactive oxygen species (ROS). Similar observations were made by Biller-Takahashi et al. [11] in pacu, whose leukocyte respiratory activity was significantly elevated in fish that received 0.3 and 1.8 mg/kg of selenium yeast and in the exotic species *Labeo rohita*, which showed a significant increase in this response after feeding with zinc nanoparticles and selenium [48].

The concentration of total proteins is primarily influenced by variations in plasma volume, with increases resulting from the shift of fluid from the plasma to the intracellular compartment [38], which was verified in this work. The osmotic imbalance generated by transport stress led to an outflow of plasma fluids from the extracellular compartments into the cells, increasing cell volume (MCV) and resulting in a higher concentration of total plasma proteins (including albumin and globulin, which are part of total proteins). Our results indicate that the electrolyte disturbances induced by transportation were more pronounced in fish supplemented with the lowest concentrations of selenium. This was demonstrated by a significant change in red blood cell volume in fish fed 0.3 mg Se/kg, as well as increased levels of albumin and globulin, which resulted in a reduced A:G ratio in fish supplemented with 0.6 mg Se/kg.

In this study, stress elevated the activity of superoxide dismutase, especially when 0.9 mg Se/kg was included. SOD is the enzyme that acts as the first antioxidant enzyme in the line of defense against reactive oxygen species, transforming the superoxide radical into hydrogen peroxide and O_2_, which may explain the activation of this enzyme 24 h after the fish have been subjected to the transport. When supplemented with 0.9 mg of Se/kg, there was an increase in the activity of this enzyme compared to before transport (basal), suggesting that this concentration of selenium in the diet may have contributed to the activation of SOD when the fish were exposed to the challenge.

CAT is an active enzyme in the peroxisomes and cytosol of cells, and perhaps 24 h may have been too short a time to verify the effect of selenium on the activation of this enzyme. However, Ferreira et al. [13] showed that one week after the fish were subjected to transport, there was a good antioxidant effect in the supplementation of 0.9 mg Se/kg, with an increase in CAT and SOD activity, allowing us to suggest that within 24 h after transport, the dose of 0.9 mg Se/kg showed promise in activating the antioxidant system.

The activation of these enzymes at higher levels corroborates several studies that demonstrate the positive effects of selenium supplementation on the antioxidant status of several fish species [49,50,51] in the presence or absence of stressors [52]. The activation of antioxidant defense parameters following stress may have contributed to the regulation of reactive oxygen species (ROS), leading to the observed decrease in leukocyte respiratory activity after management in this study. The GSH concentration and GST enzyme activity were not influenced by the stress of transportation and selenium supplementation, contrary to what was observed in *Brycon amazonicus* [53] and *Oreochromis niloticus* [54], which showed an increase in GSH concentrations and alterations in glutathione s-transferase (GST) activity after supplementation with selenium.

## 5. Conclusions

The data show that transport as an acute stressor caused a rise in blood cortisol and glucose, a reduction in the immune response (leukocyte respiratory activity), activation of the antioxidant defense system (SOD and CAT activity, mainly), and generated osmotic imbalance, leading to hypochloremia, an increase in the volume of red blood cells and a higher concentration of proteins in the plasma.

The dietary inclusion of selenium did not alleviate the hormonal, metabolic, hematological, and ionic changes caused by transport, but at a concentration of 0.9 mg Se/kg, it was effective in enhancing the activity of enzymes associated with oxidative stress, primarily superoxide dismutase, in tambaqui juveniles subjected to this challenge.

## Figures and Tables

**Table 1:** Physicochemical data of water before transport (BT), after transport (AT), and 24 h after transport (24 h AT) of tambaqui (*Colossoma macropomum*) supplemented for 75 days with diets presenting different concentrations of selenium (hydroxy-selenomethionine).

		Sampling Times		
Parameters	mg Se/kg	BT	AT	24 h AT
Dissolved oxygen (mg/L)	0.0	6.59 ± 0.44 ^A^	2.41 ± 0.97 ^B^	6.19 ± 0.15 ^A^
	0.3	6.80 ± 0.20 ^A^	2.70 ± 0.66 ^B^	6.83 ± 0.31 ^A^
	0.6	5.42 ± 0.32 ^A^	1.08 ± 0.11 ^B^	6.65 ± 0.30 ^A^
	0.9	6.25 ± 0.11 ^A^	4.82 ± 2.14 ^A^	6.04 ± 0.05 ^A^
	1.2	6.94 ± 0.47 ^A^	2.23 ± 0.73 ^B^	6.46 ± 0.31 ^A^
Temperature (°C)	0.0	29.23 ± 0.41 ^A^	27.73 ± 0.19 ^B^	26.73 ± 0.09 ^B^
	0.3	29.50 ± 0.57 ^A^	27.47 ± 0.03 ^B^	26.97 ± 0.03 ^B^
	0.6	29.44 ± 0.24 ^A^	27.57 ± 0.03 ^B^	26.70 ± 0.20 ^B^
	0.9	29.10 ± 0.06 ^A^	27.07 ± 0.39 ^B^	26.97 ± 0.03 ^B^
	1.2	29.73 ± 0.68 ^A^	27.50 ± 0.06 ^B^	26.83 ± 0.03 ^B^
pH	0.0	8.35 ± 0.02 ^A^	7.02 ± 0.07 ^B^	8.39 ± 0.02 ^A^
	0.3	8.34 ± 0.01 ^A^	7.00 ± 0.05 ^B^	8.34 ± 0.01 ^A^
	0.6	8.37 ± 0.01 ^A^	7.00 ± 0.02 ^B^	8.37 ± 0.01 ^A^
	0.9	8.34 ± 0.02 ^A^	7.05 ± 0.08 ^B^	8.34 ± 0.01 ^A^
	1.2	8.37 ± 0.01 ^A^	7.03 ± 0.06 ^B^	8.35 ± 0.01 ^A^
Alkalinity (mg CaCO_3_/L)	0.0	306.67 ± 50.34 ^A^	173.00 ± 2.08 ^B^	229.33 ± 6.69 ^AB^
	0.3	234.00 ± 15.88 ^A^	175.00 ± 0.58 ^A^	228.67 ± 3.48 ^A^
	0.6	243.33 ± 1.76 ^AB^	180.00 ± 1.53 ^B^	276.67 ± 9.68 ^A^
	0.9	260.60 ± 34.64 ^A^	176.67 ± 2.67 ^A^	251.00 ± 9.29 ^A^
	1.2	288.67 ± 28.30 ^A^	175.00 ± 2.08 ^B^	257.67 ± 12.25 ^AB^
Non-ionized ammonia (mg NH_3_/L)	0.0	0.0483 ± 0.014	0.0247 ± 0.004	0.0333 ± 0.002
	0.3	0.0360 ± 0.001	0.0236 ± 0.003	0.0306 ± 0.003
	0.6	0.0503 ± 0.012	0.0230 ± 0.001	0.0317 ± 0.003
	0.9	0.0460 ± 0.011	0.0257 ± 0.005	0.0413 ± 0.011
	1.2	0.0387 ± 0.002	0.0260 ± 0.001	0.0310 ± 0.001

Mean ± standard error. Different capital letters in the lines show significantly different data between times (ANOVA; Tukey test; *p* < 0.05). BT: before transport; AT: after transport; 24 h AT: 24 h after transport.

**Table 2:** Serum cortisol and blood glucose of tambaqui (*Colossoma macropomum*) supplemented with selenium in the diet (hydroxy-selenomethionine) and submitted to transport stress.

		Cortisol (ηg/mL)	Blood Glucose (mmol/L)
Selenium concentration (mg Se/kg diet)	0.0	61.99 ± 4.21 ^b^	6.09 ± 1.07
	0.3	74.04 ± 3.01 ^a^	4.21 ± 0.82
	0.6	68.99 ± 2.91 ^ab^	5.05 ± 0.91
	0.9	71.88 ± 3.39 ^a^	4.54 ± 1.14
	1.2	62.30 ± 3.80 ^b^	4.56 ± 1.10
Sampling times	BT	50.48 ± 3.70 ^C^	2.76 ± 0.15 ^B^
	AT	78.81 ± 1.20 ^A^	9.68 ± 0.40 ^A^
	24 h AT	62.63 ± 2.02 ^B^	1.57 ± 0.08 ^C^

Mean ± standard error. Different lower-case letters in the columns show significantly different data between treatments and capital letters between sampling times (ANOVA; Tukey test; *p* < 0.05). BT: before transport; AT: after transport; 24 h AT: 24 h after transport.

**Table 3:** Blood parameters of tambaqui (*Colossoma macropomum*) supplemented with selenium in the diet (hydroxy-selenomethionine) and submitted to transport stress.

		Hematocrit (%)	Erythrocyte Number (Cells × 10^6^/µL)	Hemoglobin Concentration (g/dL)	MCHC (g/dL)	MCH (µg)
Selenium concentration (mg Se/kg diet)	0.0	35.15 ± 1.17	1.67 ± 0.19	10.29 ± 0.48	28.09 ± 1.09	84.59 ± 15.43
	0.3	36.76 ± 0.85	1.52 ± 0.22	10.01 ± 0.54	27.18 ± 1.41	99.43 ± 19.38
	0.6	36.36 ± 0.67	1.50 ± 0.18	9.87 ± 0.44	27.70 ± 1.03	77.81 ± 11.77
	0.9	35.92 ± 1.28	1.50 ± 0.16	9.82 ± 0.60	27.22 ± 1.21	83.13 ± 13.47
	1.2	33.02 ± 1.44	1.55 ± 0.18	9.81 ± 0.54	27.74 ± 1.17	69.01 ± 10.65
Sampling times	BT	34.18 ± 0.76 ^B^	1.83 ± 0.10 ^A^	8.20 ± 0.28 ^B^	24.16 ± 0.75 ^B^	48.45 ± 3.02 ^B^
	AT	38.28 ± 0.88 ^A^	2.17 ± 0.13 ^A^	12.64 ± 0.25 ^A^	32.82 ± 0.64 ^A^	70.10 ± 5.81 ^B^
	24 h AT	33.62 ± 0.84 ^B^	0.68 ± 0.08 ^B^	8.90 ± 0.28 ^B^	25.78 ± 0.63 ^B^	150.97 ± 18.14 ^A^

Mean ± standard error, MCHC: mean corpuscular hemoglobin concentration; HCM: mean corpuscular hemoglobin (HCM). Different capital letters in the columns show significantly different data between sampling times (ANOVA; Tukey test; *p* < 0.05). BT: before transport; AT: after transport; 24 h AT: 24 h after transport.

**Table 4:** Protein profile of tambaqui (*Colossoma macropomum*) supplemented with selenium in the diet (hydroxy-selenomethionine) and submitted to transport stress.

		Total Proteins (g/dL)	Globulin (g/dL)	A:G Ratio
Selenium concentration (mg Se/kg diet)	0.0	3.98 ± 0.19	3.29 ± 0.18	1.23 ± 0.02 ^bc^
	0.3	3.73 ± 0.16	3.09 ± 0.14	1.25 ± 0.02 ^ab^
	0.6	4.02 ± 0.18	3.38 ± 0.15	1.19 ± 0.02 ^c^
	0.9	4.01 ± 0.19	3.06 ± 0.16	1.29 ± 0.02 ^a^
	1.2	3.91 ± 0.18	3.09 ± 0.15	1.20 ± 0.01 ^bc^
Sampling times	BT	3.66 ± 0.09 ^B^	3.10 ± 0.09 ^B^	1.19 ± 0.01 ^B^
	AT	4.89 ± 0.10 ^A^	3.85 ± 0.10 ^A^	1.24 ± 0.01 ^A^
	24 h AT	3.27 ± 0.06 ^C^	2.55 ± 0.06 ^C^	1.28 ± 0.02 ^A^

Mean ± standard error. Different lowercase letters in the columns show significantly different data between treatments and capital letters between sampling times (ANOVA; Tukey test; *p* < 0.05). BT: before transport; AT: after transport; 24 h AT: 24 h after transport.

## Data Availability

The data that support the findings of this study are available from the corresponding author upon reasonable request.
